# Gastrografin reduces the need for additional surgery in postoperative small bowel obstruction patients without long tube insertion: A meta‐analysis

**DOI:** 10.1002/ags3.12223

**Published:** 2018-12-17

**Authors:** Mitsuru Ishizuka, Norisuke Shibuya, Kazutoshi Takagi, Yoshimi Iwasaki, Hiroyuki Hachiya, Taku Aoki, Keiichi Kubota

**Affiliations:** ^1^ Department of Gastroenterological Surgery Dokkyo Medical University Tochigi Japan

**Keywords:** Gastrografin, meta‐analysis, postoperative small bowel obstruction, small bowel obstruction, water‐soluble contrast medium

## Abstract

**Background:**

Small bowel obstruction (SBO) is a well‐known major postoperative complication requiring immediate diagnosis and treatment to avoid additional invasive surgical procedures. Water‐soluble contrast medium is often given not only for diagnosis but also for treatment. Although numerous studies have investigated the significance of this treatment, no consensus has yet been established regarding its indications and efficacy.

**Objective:**

To explore whether Gastrografin can reduce the need for additional surgery in patients with postoperative SBO (PSBO).

**Methods:**

We carried out a comprehensive electronic search of the literature (Cochrane Library, MEDLINE, PubMed and the Web of Science) up to February 2017 to identify studies that had shown efficacy of Gastrografin in reducing the need for surgery in patients with PSBO. To integrate the individual effects of Gastrografin, a meta‐analysis was done using random‐effects models to calculate the risk ratio (RR) and 95% confidence interval (CI), and heterogeneity was analyzed using *I* ^2^ statistics.

**Results:**

Twelve studies involving a total of 1153 patients diagnosed as having PSBO were included in this meta‐analysis. Not all patients received long‐tube insertion. Among 580 patients who received Gastrografin, 100 (17.2%) underwent surgery, whereas among 573 patients who did not receive Gastrografin, 143 (25.0%) underwent surgery. Giving Gastrografin significantly reduced the need for surgery (RR, 0.66; 95% CI, 0.46‐0.95; *P* = 0.02; *I* ^2^ = 52%) in comparison with patients who did not receive Gastrografin.

**Conclusion:**

Results of this meta‐analysis show that giving Gastrografin reduces the need for surgery in PSBO patients without long‐tube insertion.

## 1 INTRODUCTION

Among several complications occurring after abdominal surgery, it is well known that small bowel obstruction (SBO) is one of the most important and common.^1^ In order to reduce the incidence of postoperative SBO (PSBO), surgeons have explored a number of options for minimizing intra‐abdominal adhesion. These have included active use of laparoscopic surgery instead of open surgery^2^ and the use of adhesion barrier film to prevent adhesion between the small bowel and the abdominal wall.^3^ However, PSBO still remains a serious problem.^4^

There are two basic types of intervention for PSBO: conventional and surgical. Conventional intervention should be undertaken as a first choice before surgical intervention because of its low degree of invasiveness.^5^ Among such interventions, giving water‐soluble contrast medium (WSCM) through a nasogastric tube (NG tube) should be carried out after immediate decompression, because this type of medium is considered to be useful for not only diagnosis^6^ but also for treatment of SBO through its osmic effect.^7^ However, although several reports have demonstrated the usefulness of this treatment for PSBO,^8^ its effects are still controversial.^8^, ^9^ In the present study, therefore, we carried out a meta‐analysis to investigate whether giving Gastrografin (Bayer Healthcare, Loos, France), a WSCM, can reduce the need for surgery in PSBO patients without long‐tube insertion.

## 2 MATERIALS AND METHODS

### 2.1 Search strategy

A systematic literature search was conducted using the Cochrane Library, MEDLINE, PubMed and the Web of Science covering papers published up to February 2017. The search was restricted to English‐language articles. Search terms used were “small bowel obstruction” and “Gastrografin”. Of those identified as potentially relevant, complete articles were retrieved and evaluated for inclusion. References from all of the relevant articles were hand‐searched for additional studies.

The meta‐analysis and search strategy complied with the guidelines of Preferred Reporting Items for Systematic reviews and Meta‐Analyses (PRISMA) 2010.^10^ Therefore, the PICO criteria for this study were: Patients (P): patients with PSBO; Intervention (I): receiving Gastrografin; Comparison (C): Control group without receiving Gastrografin; Outcome (O): surgery for PSBO.

### 2.2 Inclusion and exclusion criteria

Inclusion criteria were as follows: (i) randomized controlled trials (RCT) or other comparative studies except those with a retrospective design. (ii) Studies that provided data suitable for evaluation of PSBO. (iii) Studies that provided data allowing calculation of the risk ratio (RR) or standardized incidence ratios with 95% confidence interval (CI). (iv) Studies that provided sample size and other appropriate data. (v) Articles had to be written in English.

Exclusion criteria were: (i) Non‐reporting of predefined outcomes for two groups, such as patients with or without Gastrografin, or inability to extract the number of outcome events from the published results. (ii) Urological, gynecological and pediatric surgery, or surgery involving animal models. (iii) Articles that were letters, comments, correspondences, editorials and reviews. (iv) Studies for which the published articles had considerable overlap between authors, centers and participants. (v) Studies using Urografin instead of Gastrografin.

### 2.3 Study selection and data extraction

Full‐text reviews were carried out independently by two of the authors (M.I. and N.S.) on the basis of the inclusion and exclusion criteria and PICO. Any disagreements were resolved by discussion and consensus. The same two authors also independently extracted the following information from each eligible article: first author's name, year of publication, nation in which the study was carried out, study design, number of patients with PSBO undergoing surgery, and sample size. If the necessary data could not be extracted from the publication, we contacted the original authors directly whenever possible.

### 2.4 Data synthesis and statistical analysis

Review Manager (ver. 5.3) for Windows (downloaded from http://ims.cochrane.org/revman/download) was used for this meta‐analysis. Because there were 12 RCT, a random‐effect model was used rather than a fixed‐effect model.

Dichotomous variables were analyzed by assessing the RR of surgery in PSBO patients treated with Gastrografin compared with those who were not treated with Gastrografin as a control group, along with the 95% CI. RR of less than 1 favored patients who were treated with Gastrografin.

Statistical heterogeneity was complemented with the *I* ^2^ statistic, which qualified the proportion of total variation across studies that was due to heterogeneity rather than to chance. Presence of publication bias was assessed by funnel plot. Forest plots were demonstrated in order by weight of each study. *P* value < 0.05 was considered to indicate statistical significance.

Ethical approval was not required because this was a meta‐analysis of previously published literature.

## 3 RESULTS

### 3.1 Study identification and eligibility

**Figure 1:**
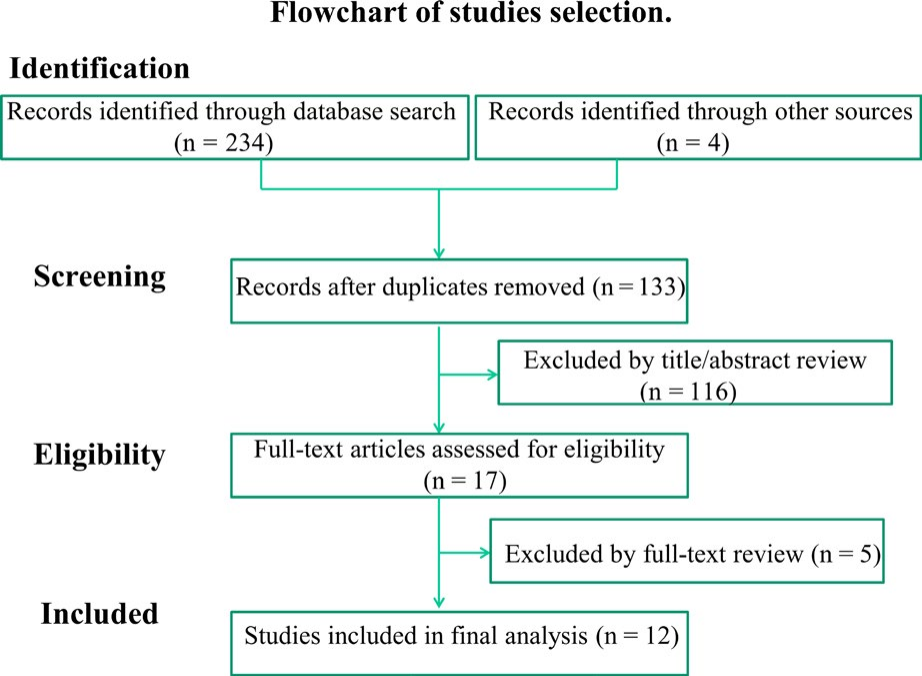
Flow diagram detailing search strategy and identification of studies used in data synthesis

An electronic search yielded 234 articles, of which 105 were regarded as duplicate articles based on a title search. Among the remaining articles, 116 were excluded by title/abstract review on the basis of their selection criteria and PICO. The remaining 17 articles were screened by full‐text review, after which 12 studies including a total of 1153 patients with PSBO were regarded as suitable for inclusion in the data synthesis.^9^, ^11^, ^12^, ^13^, ^14^, ^15^, ^16^, ^17^, ^18^, ^19^, ^20^, ^21^ The selection process for exclusion is shown in Figure 1.

### 3.2 Characteristics of included studies

All of the 12 studies were RCT. Among them, two were designed as multicenter RCT.^10^, ^18^ Basic characteristics of the 12 included studies are shown in Table 1.

**Table 1:** Summary of the 12 included studies

Author	Year	Nation	Dose of Gastrografin (mL)	Type of SBO	Type of trial	Gastrografin			Control			Study number	Control	Center	Route of administration	Gastrografin‐related complications	Timing of giving Gastrografin	Indications for surgery after giving Gastrografin	Sign of strangulation or peritonitis	Definition of PSBO
						Total	Surgery (+)	Surgery (−)	Total	Surgery (+)	Surgery (−)									
Assalia^11^	1994	Israel	100	ASBO, partial SBO	Prospective RT	59	6	53	48	10	38	None	None	Single	NG tube	None	ND (after NG tube drainage)	Continuing symptoms and signs of PSBO or clinical deterioration, coupled with persistence of radiological evidence, implied failure of conservative management and prompted laparotomy, usually not later than 48 h after admission	NR	Adhesive PSBO as defined by Brolin^31^
Feigin^12^	1996	Israel	100	PSBO	Prospective RT	25	3	22	25	4	21	None	None	Single	NG tube	None	ND (after NG tube drainage)	Surgery was done if no resolution of obstruction was achieved within 5 d	Strangulation obstruction was observed 1 (1/3) in Gastrografin and 1 (1/4) in control group, respectively	On the basis of clinical and radiological diagnosis
Fevang^13^	2000	Norway	100 + barium 100 mL	SBO	Prospective RT	48	17	31	50	15	35	None	None	Single	NG tube or orally	NR	ND (after NG tube drainage)	Surgery was done if obstruction did not resolve spontaneously	Strangulation obstruction was observed 1 (1/48) in Gastrografin and 4 (4/50) in control group, respectively	Judged by plain abdominal radiograph
Biondo^14^	2003	Spain	100	ASBO	Prospective RT	44	5	39	46	8	38	None	None	Single	NG tube	NR	After complete suction of gastric fluid	Surgery was done if there was no clinical or radiological improvement in the following 24 h	NR	On the basis of a clinical picture of abdominal pain, distension, vomiting and abnormal bowel sounds
Burge^15^	2005	New Zealand	100	ASBO	RCT	22	4	18	21	4	17	None	Isotonic saline	Single	NG tube	None	As soon as after stomach emptying	Patients with adhesive SBO having any complications including strangulation by 4 d underwent surgery	NR	On the basis of clinical and radiological evidence
Zhang^16^	2006	China	100	ASBO	RT	80	7	73	82	21	61	None	None	Single	NG tube	NR	After complete suction of gastric fluid	Laparotomy was done if symptoms of strangulation developed or if the obstruction did not resolve spontaneously after 3 d	NR	On the basis of clinical symptoms of abdominal pain, abnormal bowel sounds, distension and vomiting, and radiological findings of dilated small bowel loops
Di Saverio^17^	2008	Italy	150 + 50 mL water	ASBO	Prospective RCT	38	7	31	38	17	21	NCT00601809	None	Multi	NG tube	None (only 3 patients vomited)	ND (after NG tube drainage)	If the contrast did not reach the colon after 36 h, subjects underwent laparotomy	Strangulation obstruction was observed 1 (1/7) in Gastrografin and 2 (2/17) in control group, respectively	On the basis of clinical and radiological evidence of PASBO
Kumar^18^	2009	India	60	PSBO	Prospective RT	21	7	14	20	2	18	None	None	Single	NG tube	NR	After decompression of the stomach was done	Persistence of SBO for 48 h after admission or clinical deterioration with persistence or worsening of radiological evidence during the in‐hospital course	NR	On the basis of clinical history, examination and abdominal radiograph findings
Farid^19^	2010	Egypt	100	ASBO	Prospective RT	55	8	47	55	19	36	None	None	Single	NG tube	NR	After complete suction of gastric fluid	Patients in whom abdominal radiography with Gastrografin failed to reach the colon after 24 h were subjected to surgical exploration	Among 126 patients, 4 patients were excluded from the study because of strangulation. In addition, strangulation obstruction was observed 2 (2/8) in Gastrografin and 3 (3/19) in control group, respectively	On the basis of clinical and radiological pictures of PASBO
Rahmani^20^	2013	Iran	100	PASBO	Prospective RT	42	4	38	42	10	32	None	None	Single	NG tube	NR	ND (after NG tube drainage)	Patients who showed no progressive clinical and radiological improvement after 4 d underwent surgery.	NR	On the basis of clinical and radiological pictures of PASBO
Haule^21^	2013	Uganda	100 (60 mL 5‐10 y children)	ASBO	Open RCT	25	3	22	25	9	16	None	None	Single	NG tube	NR	ND (after NG tube drainage)	Patients who did not show improvement within a maximum of 5 d underwent surgery.	NR	On the basis of clinical features referring to symptoms, signs and radiological evidence of ASBO
Scotte^9^	2017	France	100	ASBO	RT	121	29	92	121	24	97	NCT00389116	0.9% NaCl solution	Multi	NG tube	NR	After 2 h of nasogastric aspiration	If neither flatus nor accumulation of contrast in the cecum was observed after 48 h, decision to operate was taken	Radiological signs of peritonitis or strangulation were defined as exclusion criteria	On the basis of CT of the abdomen consistent with an uncomplicated ASBO

ASBO, adhesive small bowel obstruction; CT, computed tomography; ND, not defined; NG tube, nasogastric tube; NR, not reported; PASBO, postoperative adhesive small bowel obstruction; PSBO, postoperative small bowel obstruction; RCT, randomized controlled trial; RT, randomized trial; SBO, small bowel obstruction.

### 3.3 Association between giving Gastrografin and surgery for PSBO

Data on surgery for PSBO were available for all 12 RCT.

With regard to the dose of Gastrografin given, 10 studies recommended 100 mL. Among them, one study recommended 60 mL Gastrografin for pediatric patients^21^ and one study added 100 mL barium to 100 mL Gastrografin.^13^ One study recommended 150 mL Gastrografin^17^ and one study recommended 60 mL Gastrografin.^18^ No Gastrografin‐related complications (eg, fluid or electrolyte disturbance, aspiration pneumonia, or exacerbation of obstructive episodes)^11^ were reported. In fact, previous studies have shown that complications, including allergic reactions, resulting from the use of Gastrografin are rare.^22^

In all 12 RCT, Gastrografin was given through a NG tube. In one study, the timing of dosage was defined as after 2 hours of NG‐tube aspiration;^9^ in the other 11 studies, the timing of Gastrografin dosage by a NG tube was not clearly stated. Not all patients received long‐tube insertion.

Indications for surgery after receiving Gastrografin are shown in Table 1. Patients who were and who were not given Gastrografin were considered to require surgery if features of strangulation or peritonitis appeared during the in‐hospital course.

**Figure 2:**
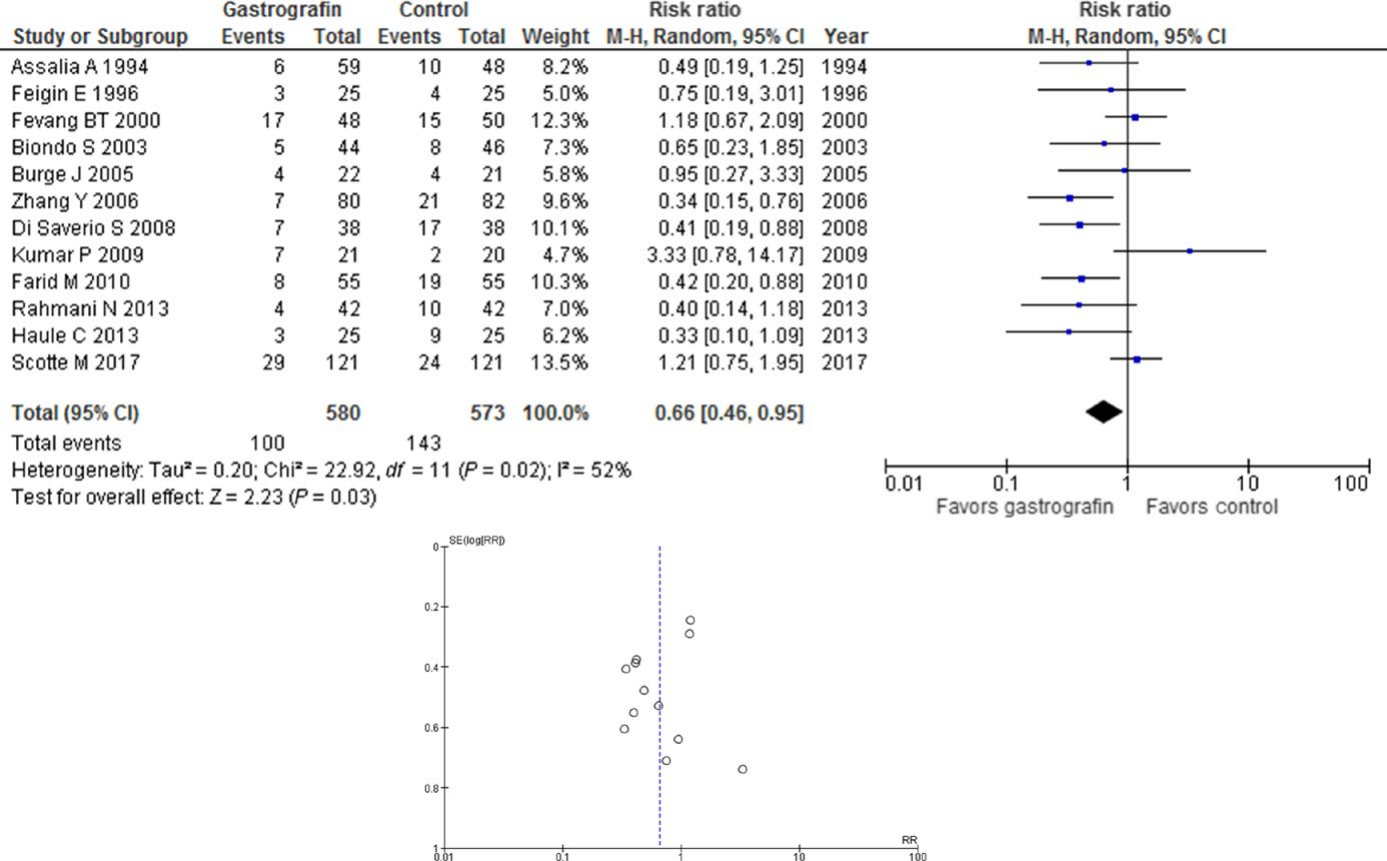
Forest plot of the occurrence of surgery for patients with postoperative small bowel obstruction and funnel plot analysis of such patients using integrated data

Among 580 patients who received Gastrografin, 100 (17.2%) underwent surgery, whereas among 573 patients who did not receive Gastrografin, 143 (25.0%) underwent surgery. Giving Gastrografin significantly reduced the need for surgery for PSBO (RR, 0.66; 95% CI, 0.46‐0.95; *P *=* *0.03; *I* ^2^ = 52%) in comparison with patients who did not receive Gastrografin (Figure 2).

The basic funnel plot of the studies included in this meta‐analysis indicated no evidence of publication bias, in view of its symmetry (Figure 2).

## 4 DISCUSSION

A systematic literature search has shown that two types of WSCM are used: Gastrografin and Urografin (Bayer Healthcare).^23^ In fact, several previous studies that investigated the usefulness of this type of medium for PSBO included both types. However, for the present study, we selected Gastrografin only, a mixture of sodium diatrizoate and meglumine diatrizoate, instead of Urografin, because Urografin is not used as a contrast medium for the gastrointestinal tract in Japan. In terms of their components, there is, in fact, no difference between Gastrografin (sodium diatrizoate 59.73 g and meglumine diatrizoate 15.924 g in 100 mL) and 76% Urografin (sodium diatrizoate 59.73 g and meglumine diatrizoate 15.924 g in 100 mL). In fact, although Gastrografin is generally used for gastrointestinal studies, Urografin is used as a contrast agent for direct pancreatic duct cholangiography, retrograde urography, arthrography and sialography. Therefore, we extracted Urografin from this study.

Theoretically, giving Gastrografin is recommended for conservative treatment of patients with PSBO before surgery because it has a very high osmolality and acts by drawing water into the lumen of the small bowel, thus reducing small bowel wall edema and assisting recovery of bowel motility.^5^ Although only one small prospective randomized trial (RT) has investigated the therapeutic value of Gastrografin in 19 patients with adhesive SBO after unsuccessful conservative treatment, the authors concluded that its use in this setting was safe and significantly reduced the need for surgery by 74%.^7^

However, the results of four previous meta‐analyses that investigated the significance of WSCM administration for patients with PSBO were somewhat controversial.^9^, ^24^, ^25^, ^26^ Although two of the meta‐analyses concluded that giving WSCM reduced the need for surgery in patients with adhesive SBO,^25^, ^26^ the other two studies concluded that WSCM did not reduce the need for surgery in such patients.^9^, ^24^ One^24^ of the latter two studies was the earliest among the four, and included the lowest number of both patients and PSBO studies. Similarly, the other^9^ of the latter two studies, which was the latest of the total of four meta‐analyses, included the largest number of patients with PSBO among the 10 included studies.

Moreover, in comparison with the four previous meta‐analyses,^9^, ^24^, ^25^, ^26^ our present findings clearly confirmed the usefulness of Gastrografin for reducing the need for surgery, especially as it was based on the largest number of both patients and RCT. Our analysis did not include any study that had used Urografin as WSCM for treatment of patients with PSBO. In fact, among the 12 RCT, three clearly indicated the usefulness of Gastrografin for treatment of PSBO.^16^, ^17^, ^19^ Furthermore, none of the analyzed RCT clearly contraindicated Gastrografin for treatment of such patients. Therefore, our present analysis has been able to provide new evidence for the utility of Gastrografin based on the four previous meta‐analyses.^9^, ^24^, ^25^, ^26^

Although the forest plot of Kumar et al^18^ seemed to show an opposite effect, they concluded that giving an oral water‐soluble contrast agent in PSBO helped with earlier resolution of obstruction and decreased the length of hospital stay. In fact, 14 (66.7%) patients had relief of obstruction after receiving the contrast material, and mean time for relief of obstruction was 7.47 hours in group A (21 patients were given an oral water‐soluble contrast agent: Gastrografin group). In contrast, 18 (90%) patients had relief of obstruction and the time interval was 35.20 hours (*P* < 0.001) in group B (20 patients were managed conventionally: control group). Mean length of hospital stay was 3.43 ± 1.08 days for group A and 5.33 ± 2.95 days for group B (*P* = 0.029). Although seven patients in group A and two in group B were operated, there was no significant difference between the two groups (*P* = 0.71).

Unlike the situation in most western countries, long‐tube insertion is generally carried out in Japan to treat patients with PSBO.^27^ Because only one small prospective RT of short versus long‐tube insertion for adhesive SBO showed no significant therapeutic difference between the two as a conventional therapy,^28^ this may explain why short‐tube insertion has commonly been recommended in western countries. In fact, in all of the RCT we analyzed, Gastrografin was given by NG tube, not by long tube.

Recent studies have shown that long tube decompression is successful in 90% of patients with adhesive SBO.^29^ For example, in every hospital in PA, USA, the standard use of improved long tube and gastrofiber scopes has increased the success rate of insertion to the small bowel to 90%, and most patients in whom decompression using short‐tube insertion fails become candidates for long‐tube insertion.^30^ Thus, currently, long‐tube insertion is strongly recommended because it provides significant clinical and economic advantages over short‐tube insertion.^30^

It is obvious that a short tube cannot sufficiently reduce intra‐small bowel pressure because the tube tip is located in the stomach. However, it is clear that a long tube can more effectively reduce intra‐small bowel pressure because the tube tip is located in the dilated small bowel and can effectively aspirate the accumulated intestinal fluid. Furthermore, the balloon of the long tube is able to assist insertion of the tube to the far distal side of the small bowel, beyond the obstructed portion.

In fact, even if PSBO patients with a short NG tube receive WSCM, effect of the WSCM is diluted by accumulated intestinal fluid in the dilated small bowel. However, if PSBO patients receive WSCM by a long tube, the WSCM can work more effectively in the decompressed small bowel or near the obstructed portion.

Although a prospective RCT comparing short‐tube versus long‐tube insertion would be required to adequately assess the effect of Gastrografin in PSBO patients, the results of this meta‐analysis clearly demonstrate that giving Gastrografin reduces the need for surgery in PSBO patients without long‐tube insertion.

## CONFLICTS OF INTEREST

Authors declare no conflicts of interest for this article.
